# Bis(dipyrrinato)metal(ii) coordination polymers: crystallization, exfoliation into single wires, and electric conversion ability†
†Electronic supplementary information (ESI) available: Experimental methods; XPS of **M1**, **Mono1**, and **Zn2**; photographs of the single crystals of **Ni1** and **Cu1**; single-crystal XRD data for **M1**; photograph of a free-standing film of **Zn1-SWCNT**; thermoelectric conversion of **Zn1-SWCNT**; photoelectric conversion and its quantitative analysis of **Zn1**, **Zn2**, and **Mono3**; photoelectric conversion setup. CCDC 1012353, 1044669 and 1044670. For ESI and crystallographic data in CIF or other electronic format See DOI: 10.1039/c5sc00273g



**DOI:** 10.1039/c5sc00273g

**Published:** 2015-02-26

**Authors:** Ryota Matsuoka, Ryojun Toyoda, Ryota Sakamoto, Mizuho Tsuchiya, Ken Hoshiko, Tatsuhiro Nagayama, Yoshiyuki Nonoguchi, Kunihisa Sugimoto, Eiji Nishibori, Tsuyoshi Kawai, Hiroshi Nishihara

**Affiliations:** a Department of Chemistry , Graduate School of Science , The University of Tokyo , 7-3-1, Hongo , Bunkyo-ku , Tokyo 113-0033 , Japan . Email: sakamoto@chem.s.u-tokyo.ac.jp ; Email: nisihara@chem.s.u-tokyo.ac.jp; b Graduate School of Materials Science , Nara Institute of Science and Technology (NAIST) , 8916-5 Takayama, Ikoma , Nara 630-0192 , Japan; c Japan Synchrotron Radiation Research Institute (JASRI) , 1-1-1, Kouto, Sayo-cho , Sayo-gun , Hyogo 679-5198 , Japan; d Division of Physics , Faculty of Pure and Applied Sciences , Tsukuba Research Center for Interdisciplinary Materials Science (TIMS) , and Center for Integrated Research in Fundamental Science and Engineering (CiRfSE) , University of Tsukuba , 1-1-1 Tennodai , Tsukuba , Ibaraki 305-8571 , Japan

## Abstract

One-dimensional coordination polymers (1D-CPs) tend either to dissociate into constitutive ligands and metals readily in solution, or to aggregate randomly and amorphously, which prevents them from widespread application. In the present research, 1D-CPs comprising bridging dipyrrin ligands and divalent metal ions (Zn^2+^, Ni^2+^, and Cu^2+^) are synthesized. A liquid/liquid interfacial reaction gives rise to single crystals suitable for X-ray diffraction analysis: A dichloromethane solution of the ligand is layered with aqueous metal(ii) acetate, such that the coordination reaction proceeds at the liquid/liquid interface. Isolated single fibers of the zinc coordination polymer may be exfoliated from the single crystal or bulk solid upon ultrasonication. Atomic force microscopy (AFM) detects the isolated fibers with lengths of more than several μm. The exfoliated 1D-CP wires feature good processability, realizing a conjugate with single-wall carbon nanotubes (SWCNTs), and a thin film on a transparent SnO_2_ electrode. The processed materials show electric conversion ability: For example, the modified SnO_2_ electrode serves as a photoanode for a photoelectric conversion system. The designability and tunability of the present 1D-CPs is demonstrated by a ligand modification, affording a luminescent property and an extension of the photoelectric conversion response to longer wavelengths.

## Abstract 2

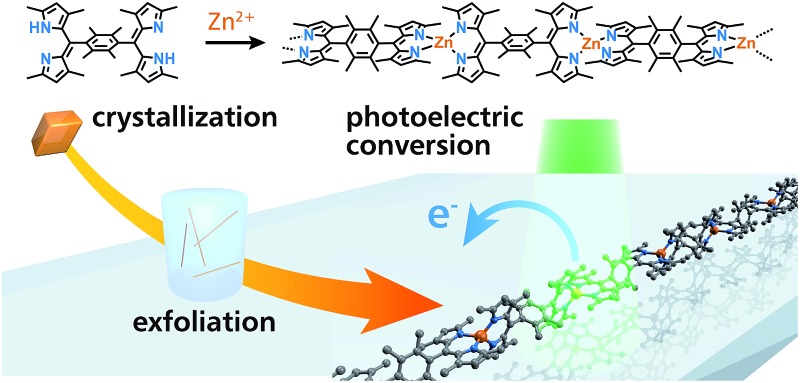
The titled coordination polymers feature crystallization, single wire exfoliation, processability, and applicability to photoelectric and thermoelectric conversion systems.

## Introduction

Two- and three-dimensional coordination polymers such as nanosheets,^1–10^ metal–organic frameworks (MOFs),^11–13^ and porous coordination polymers (PCPs)^14–16^ have attracted intense interest. On the other hand, the physical flexibility of one-dimensional coordination polymers (1D-CPs) makes them useful for conjugation with micro and nano-sized functional materials such as carbon nanotubes,^17–19^ although difficulties in handling have prevented 1D-CPs from widespread application. Many 1D coordination chains are stable only in the solid phase, and they dissociate into constitutive ligands and metals readily in solution. Furthermore, 1D-CPs tend to aggregate randomly and amorphously, and there are few examples of 1D-CPs where both crystalline phase and isolated single chain phase coexist.^20,21^

The dipyrrin-metal complex is an attractive molecular motif for coordination polymers. Dipyrrin ligands accept various metal ions, and in most cases the complexation reaction proceeds spontaneously even in the absence of a base.^22,23^ This feature is desirable for synthesizing supramolecules^24,25^ and coordination polymers.^26–28^ However, no 1D-CP based on the dipyrrin–metal complex has demonstrated crystallinity, single-chain isolation, and potential applicability at the same time.

**Fig. 1:**
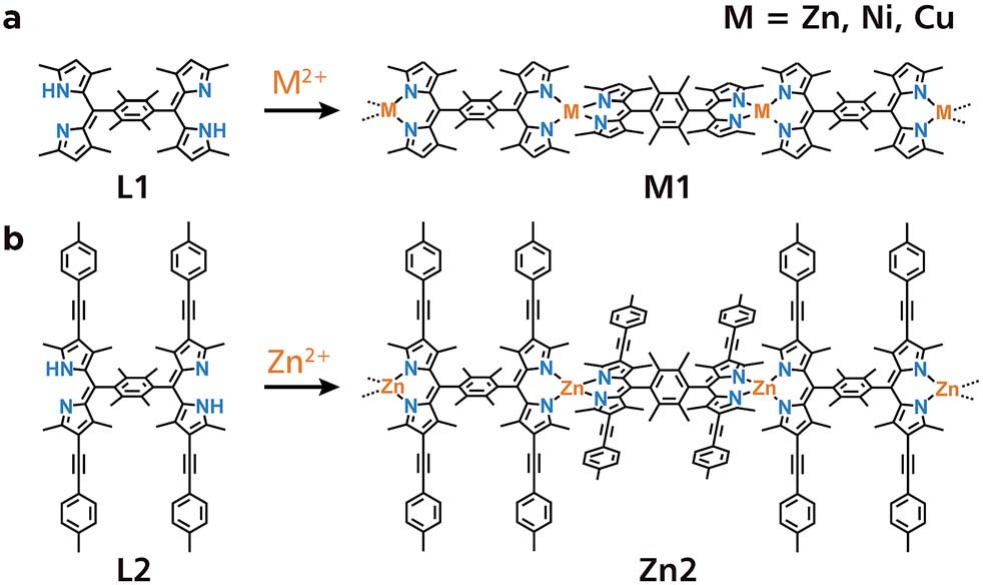
Chemical structures of bridging dipyrrin ligands and corresponding 1D-CPs based on bis(dipyrrinato)metal(ii) complexes. (a) **L1** and **M1** (**M** = Zn, Ni, Cu). (b) **L2** and **Zn2**.

In the present work, we synthesize 1D-CP **M1** featuring the bis(dipyrrinato)metal(ii) complex motif, which is composed of bridging dipyrrin ligand **L1** and divalent metal ion **M^2+^** (**M** = Zn, Ni, and Cu, Fig. 1a). A liquid/liquid interfacial reaction is effective for ordering **M1**, giving rise to single crystals suitable for X-ray diffraction analysis (XRD). Single fibers of **Zn1** are isolated from the single crystal upon ultrasonication, and then visualized by atomic force microscopy (AFM). The dispersibility of the exfoliated fibers of **Zn1** affords good processability, giving rise to a conjugate with single-wall carbon nanotubes (SWCNTs), and a thin film of **Zn1** on a transparent SnO_2_ electrode. These processed materials may be applied in thermo and photoelectric conversion systems, thereby demonstrating the utility of **Zn1**. The designability and tunability of the present 1D-CP system are illustrated with analogue **Zn2** comprising π-extended ligand **L2** (Fig. 1b), which shows luminescence in the exfoliated fibrous form, and an extension of the photoelectric conversion response to longer wavelengths.

## Results and discussion

**Fig. 2:**
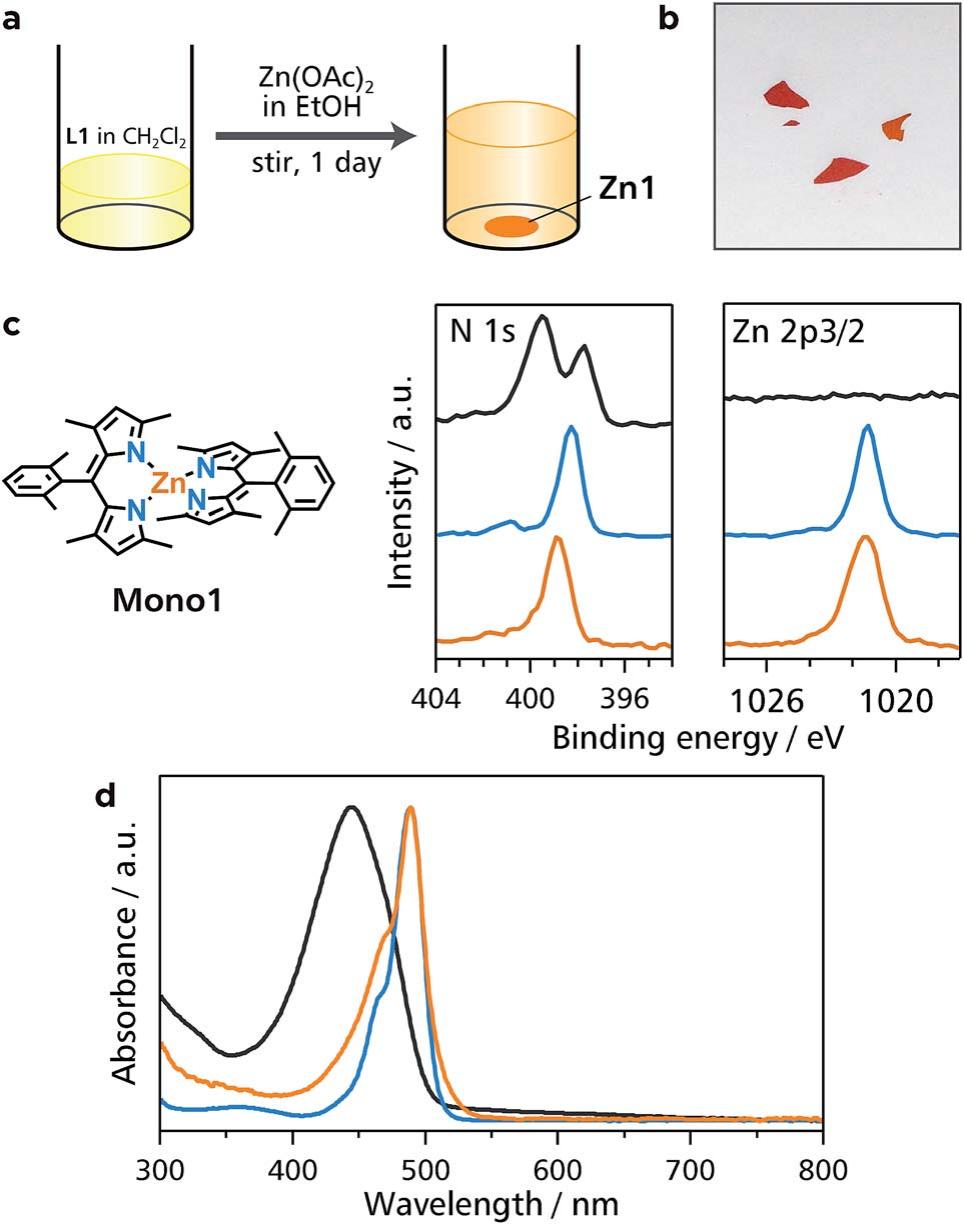
Single-phase synthesis and characterization of **Zn1**. (a) Schematic illustration of the synthesis. (b) Photograph of powdery **Zn1**. (c) Chemical structure of referential compound **Mono1**, and XPS of **L1** (black), **Mono1** (blue), and **Zn1** (orange) focusing on the N 1s and Zn 2p 3/2 regions. (d) UV/vis spectra of **L1** (black), **Mono1** (blue), and **Zn1** (orange) in DMF.

Initially, a conventional single-phase reaction was performed to synthesize **Zn1** (Fig. 1a). Equimolar amounts of bridging dipyrrin ligand **L1** and zinc(ii) acetate were reacted in a mixture of dichloromethane and ethanol (Fig. 2a and b). The resultant dark-orange powder was subjected to X-ray photoelectron spectroscopy (XPS) using **L1** and mononuclear bis(dipyrrinato)zinc(ii) complex **Mono1** as references (Fig. 2c). The Zn 2p 3/2 peak is only visible in **Mono1** and **Zn1**, which is consistent with the presence of Zn ions. Another distinctive fingerprint of the coordination between the dipyrrin ligand and zinc(ii) ions occurs in the N 1s region. Free base **L1** features two peaks (397.5 and 399.1 eV) arising from the iminic and pyrrolic nitrogens that are chemically different from one another.^29^ In contrast, **Mono1** shows a single N 1s peak, which stems from the homogenization of the two nitrogen atoms upon coordination to the zinc center. A solitary N 1s peak is also observed for **Zn1**. In addition, the nitrogen-to-zinc abundance ratio calculated from the peak area corrected by the photoionization cross-section is consistent with the ideal value of N : Zn = 4 : 1 (79.8 : 20.2 and 79.5 : 20.5 for **Zn1** and **Mono1**, respectively, Fig. S1a and b†). These results indicate the formation of the desired coordination polymer **Zn1** consisting of the bis(dipyrrinato)zinc(ii) complex. The authors note that **Ni1** and **Cu1** synthesized by means of the single-phase method also displayed nitrogen-to-metal abundance ratios of *ca.* 4 : 1 in XPS (80.2 : 19.8 and 80.3 : 19.7 for **Ni1** and **Cu1**, respectively, Fig. S1c and d†).

**Zn1** is dispersible in dimethylformamide, and was characterized by UV/vis spectroscopy (Fig. 2d). An intense absorption with a maximum at 445 nm in **L1** stems from the ^1^π–π* transition of the free-base dipyrrin moiety.^30^ However, the ^1^π–π* band is red-shifted by 44 nm in **Mono1** (489 nm), which is typical of zinc(ii) complexation with a dipyrrin ligand.^30^ The ^1^π–π* band of **Zn1** has the same absorption maximum (489 nm) as that of **Mono1**, which also shows that **Zn1** formed the bis(dipyrrinato)zinc(ii) coordination polymer.

**Fig. 3:**
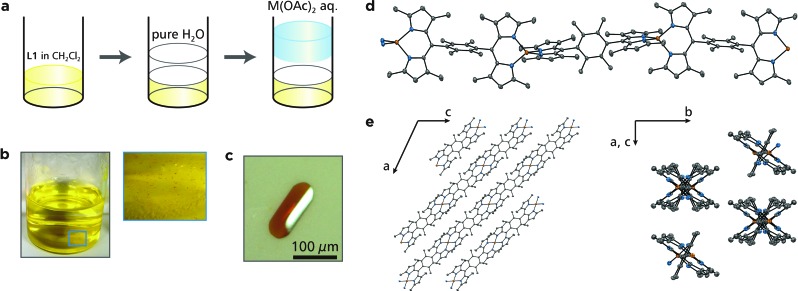
Liquid/liquid interfacial synthesis for **M1** and crystal structure of **Zn1**. (a) Schematic illustration of the liquid/liquid interfacial synthesis for **M1**. (b) (left) Photograph of the reaction system after the emergence of single crystals of **Zn1** and (right) close-up image at the liquid/liquid interface (blue square). (c) Photograph of a typical single crystal of **Zn1**. (d) ORTEP drawing of **Zn1** with a thermal ellipsoid set at the 50% probability level (C, gray; N, blue; Zn, orange). Hydrogen atoms are omitted for clarity. (e) Perspective views of 3D packing of **Zn1** along the b (left) and [1 0 –1] (right) crystallographic axes, respectively.

We have demonstrated previously that liquid/liquid interfacial synthesis—where organic ligand molecules in an organic solvent and aqueous metal ions are layered to form a liquid/liquid interface—is effective for synthesizing low-dimensional CPs with ordered secondary structures.^4,5,31,32^ For example, a liquid/liquid interfacial synthesis using 1,2,4,5-benzenetetrathiol and nickel(ii) ions produced a nickel bis(dithiolene) 1D-CP that aligns to form two-dimensional ordered structures.^31^ In the present work, the coordination reaction between bridging dipyrrin ligand **L1** in dichloromethane and divalent metal ions **M^2+^** (**M** = Zn, Ni, and Cu) in water was carried out at the liquid/liquid interface, and it produced crystals of **M1** that floated on the interface or sank to the bottom of the reaction container (Fig. 3a and b). A photograph of a typical single crystal for **Zn1** is shown in Fig. 3c, and those for **Ni1** and **Cu1** are placed in Fig. S2.† This series of crystals was analyzed by synchrotron radiation X-ray diffraction (Fig. 3d and e and S3,† and Table S1† for **Zn1**, Fig. S3 and Table S2† for **Ni1**, and Fig. S3 and Table S3† for **Cu1**).^33^ The crystal structures are almost identical to each other for the three metal centers. They show the desired 1D polymeric chains, propagating along the [1 0 –1] crystallographic axis. The metal centers adopt slightly distorted tetrahedral coordination spheres with dihedral angles of 81.29°, 81.11°, and 87.36° for Zn, Ni, and Cu, respectively. The average metal–nitrogen bond lengths are, respectively, 1.9181, 1.9151, and 1.9032 Å for Zn, Ni, and Cu. This series of dihedral angles and metal–nitrogen bond lengths are typical of bis(dipyrrinato)metal(ii) complexes bearing α-methylated dipyrrin ligands.^30,34^ Divalent nickel and copper generally prefer square-planar coordination spheres, though, the methyl group at the α-position induces tetrahedral coordination spheres even to the Ni and Cu centers because of steric hindrance. The dipyrrinato ligand is orthogonal to the bridging 2,3,4,5-tetramethylphenyl group with dihedral angles of 101.18°, 100.02°, and 98.93° for Zn, Ni, and Cu, respectively, reflecting steric hindrance between the two moieties.

**Fig. 4:**
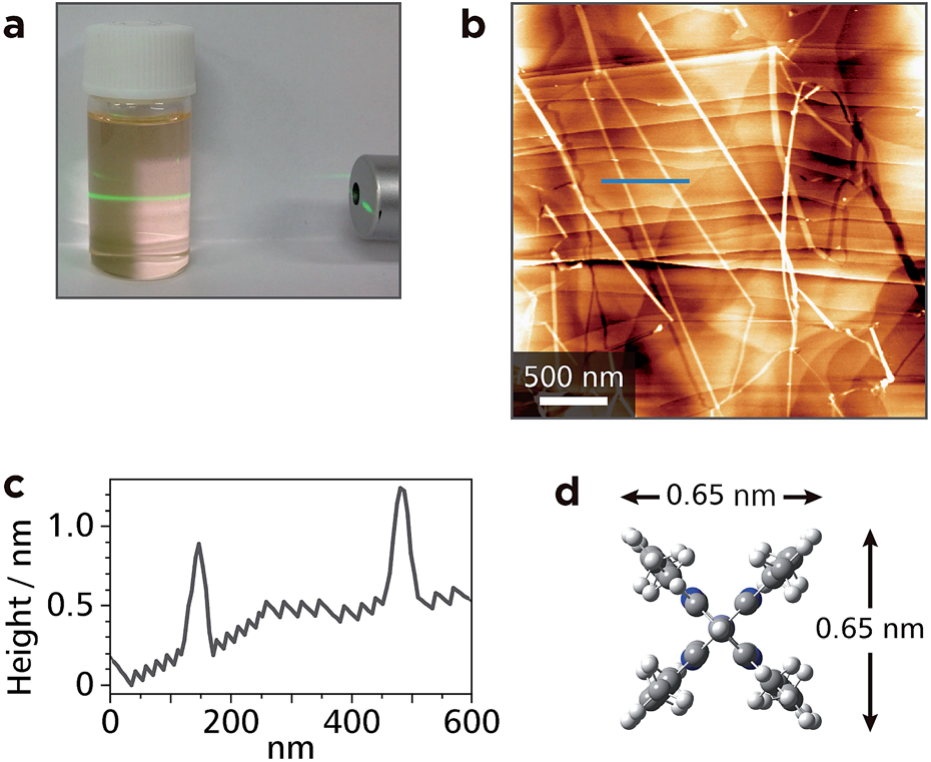
Exfoliation of single fibers of **Zn1**. (a) Tyndall scattering from a dichloromethane colloidal suspension of **Zn1** upon illumination with a green luminous flux. (b) AFM topographic image of exfoliated fibers of **Zn1** on HOPG. (c) AFM cross-sectional profile along the blue line in (b). (d) Molecular size of **Mono1** estimated by means of DFT calculation.

Upon ultrasonication, the single crystal of **Zn1** could be dispersed in dichloromethane, and showed Tyndall scattering (Fig. 4a). The dispersion was cast onto highly ordered pyrolytic graphite (HOPG), which was then subjected to AFM. A representative height image shows several straight white lines with lengths of >3 μm traversing the steps of the HOPG substrate (Fig. 4b). The cross-sectional analysis shows that the height of the lines is *ca.* 0.7 nm (Fig. 4c), which is consistent with the size of **Mono1** estimated by means of DFT calculation (Fig. 4d). This indicates that **Zn1** is stable enough both chemically and physically to be isolated as single chains. We note that the width of **Zn1** is overestimated by AFM. This is common in 1D polymer systems and AFM resolution.^35–37^

**Fig. 5:**
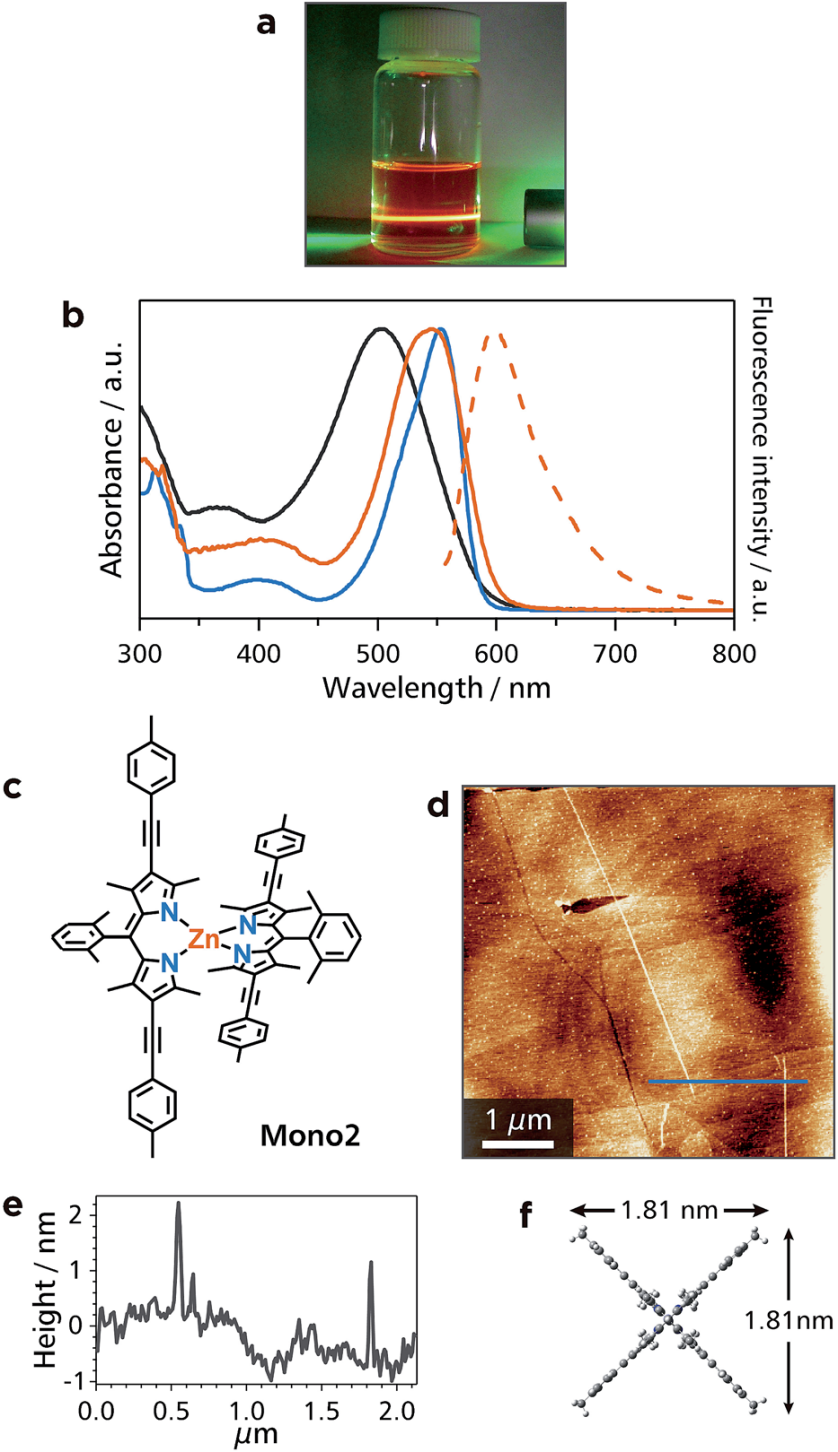
Characterization of **Zn2**. (a) Dichloromethane dispersion of **Zn2** irradiated with a green laser. Tyndal scattering was concealed by orange fluorescence from **Zn2**. (b) Absorption (solid lines) and fluorescence (dotted line) spectra of **L2** (black), **Mono2** (blue), and **Zn2** (orange) in toluene. (c) Chemical structure of referential mononuclear zinc(ii) complex **Mono2**. (d) AFM topographic image of HOPG modified with a dispersion of **Zn2**. (e) AFM cross-sectional profile along the blue line in (d). (f) Molecular size of **Mono2** estimated by means of DFT calculation.

Among bis(dipyrrinato)metal(ii) complexes, zinc-centered ones are known to possess luminescence ability. Nevertheless, **Zn1** did not show detectable fluorescence at room temperature. This situation was improved by one of the virtues of 1D-CPs, designability and tunability: The authors also synthesized π-extended bridging dipyrrin ligand **L2**, and prepared another 1D-CP **Zn2** (Fig. 1b). In XPS, **Zn2** showed a nitrogen-to-metal abundance ratio of 79.4 : 20.6, almost consistent with the ideal value (4 : 1) (Fig. S1e†). Thanks to the (4-methylphenyl)ethynyl group at the β-position of the dipyrrin ligand, the dispersibility of **Zn2** was better than that of **Zn1**. In addition, **Zn2** featured orange emission in a dichloromethane dispersion, so that Tyndall scattering was concealed (Fig. 5a). In UV/vis and luminescence spectroscopy in toluene (Fig. 5b), **Zn2** featured the ^1^π–π* absorption with a maximum at 547 nm, which is similar to that of corresponding mononuclear complex **Mono2** (*λ*_max_ = 553 nm, Fig. 5b and c). These bands are red-shifted relative to that of **L2** (*λ*_max_ = 505 nm), indicative of the complexation with zinc(ii) ions. In contrast to non-fluorescent **Zn1**, the fluorescence of **Zn2** was observed at the maximum wavelength of 597 nm (quantum efficiency: 8%): therefore, the fluorescence ability revived upon the ligand modification in **L2**. AFM for a HOPG substrate modified with a dispersion of **Zn2** disclosed straight white lines with a length of more than 4.5 μm (Fig. 5d and e). The height of the lines (1.9 nm) is in good agreement with the size of **Mono2** estimated by DFT calculation (Fig. 5f).

**Fig. 6:**
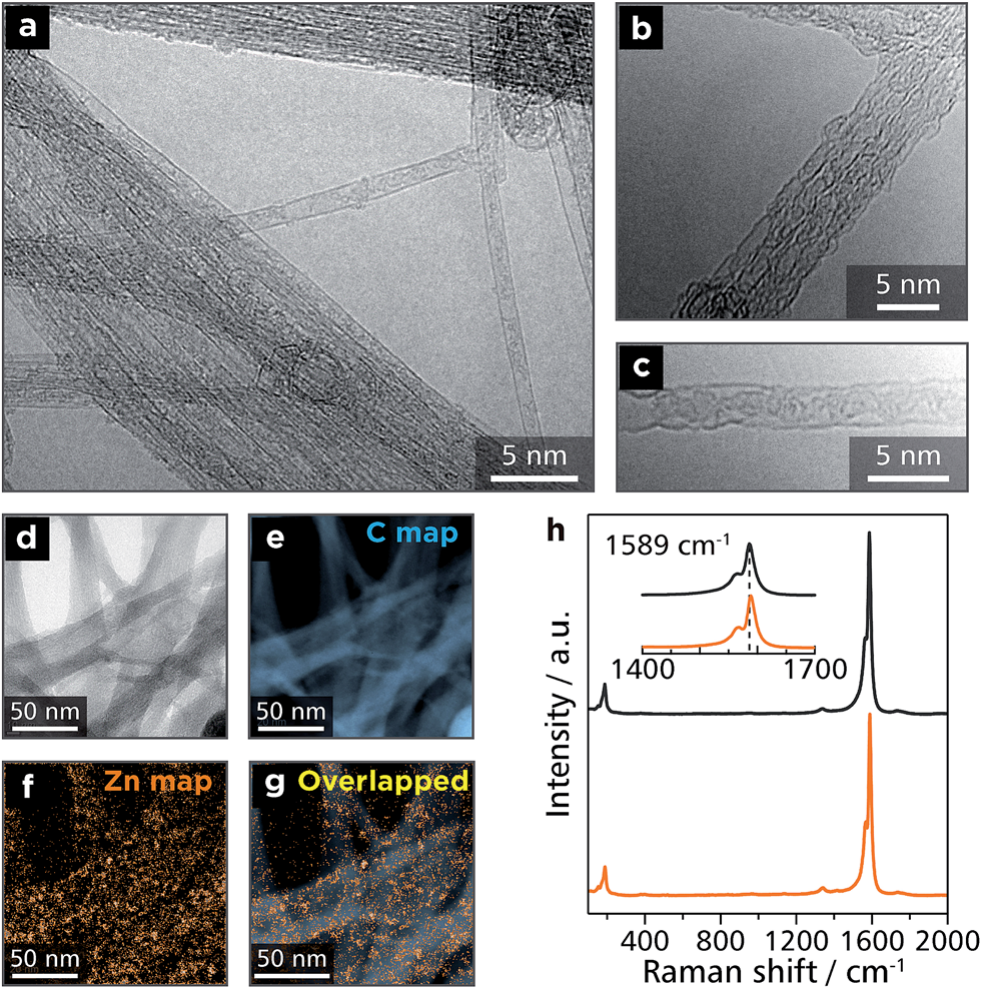
Microscopic and spectroscopic analyses for **Zn1-SWCNT**. (a)–(c) Transmission electron microscopy (TEM) images of **Zn1-SWCNT** on a Cu grid. (d) Bright-field-TEM image of **Zn1-SWCNT** subjected to electron energy-loss spectroscopy (EELS) mapping. EELS mapping for: (e) carbon K edge intensity and (f) zinc M2 and M3 edge intensities. (g) Overlapped image of (e) and (f). (h) Raman spectra of pristine SWCNTs (black) and **Zn1-SWCNT** (orange).

In order to demonstrate the processability of **Zn1**, the authors fabricated a conjugate with SWCNTs (**Zn1-SWCNT**). A mixture of **Zn1** and SWCNTs with a weight ratio of 1 : 10 was dispersed in DMF by ultrasonication for 90 min, and the dispersion was then shaken for 1 d. This process resulted in the disappearance of the orange color of **Zn1** from DMF, which indicated that **Zn1** was adsorbed onto SWCNTs. Upon filtration, the conjugate spontaneously assembled into a free-standing membrane with a thickness of 64 μm (Fig. S4†). The conjugate was then subjected to spectroscopic analyses. Fig. 6a–c shows TEM images of bundles of SWCNTs with some exfoliated tubes. On the other hand, electron energy-loss spectroscopy (EELS) reveals the presence of zinc, which is scattered uniformly across the carbon scaffold derived from the SWCNT skeleton (Fig. 6d–g). Raman spectroscopy shows an intense G band (1590 cm^–1^) and a weak D band (1350 cm^–1^), which stem from intact and damaged SWCNTs, respectively (Fig. 6h).^38^ These results indicate that **Zn1** wraps around the SWCNT uniformly without destroying the SWCNT structure. A preliminary experiment disclosed the thermoelectric conversion ability of **Zn1-SWCNT** (Fig. S5†). **Zn1-SWCNT** has a power factor of 33 μW m^–1^ K^–2^, which is greater than that of pristine SWCNTs (9.3 μW m^–1^ K^–2^), and those derived from conjugates between SWCNTs and small organic molecules (26 μW m^–1^ K^–2^ at most).^39^

**Fig. 7:**
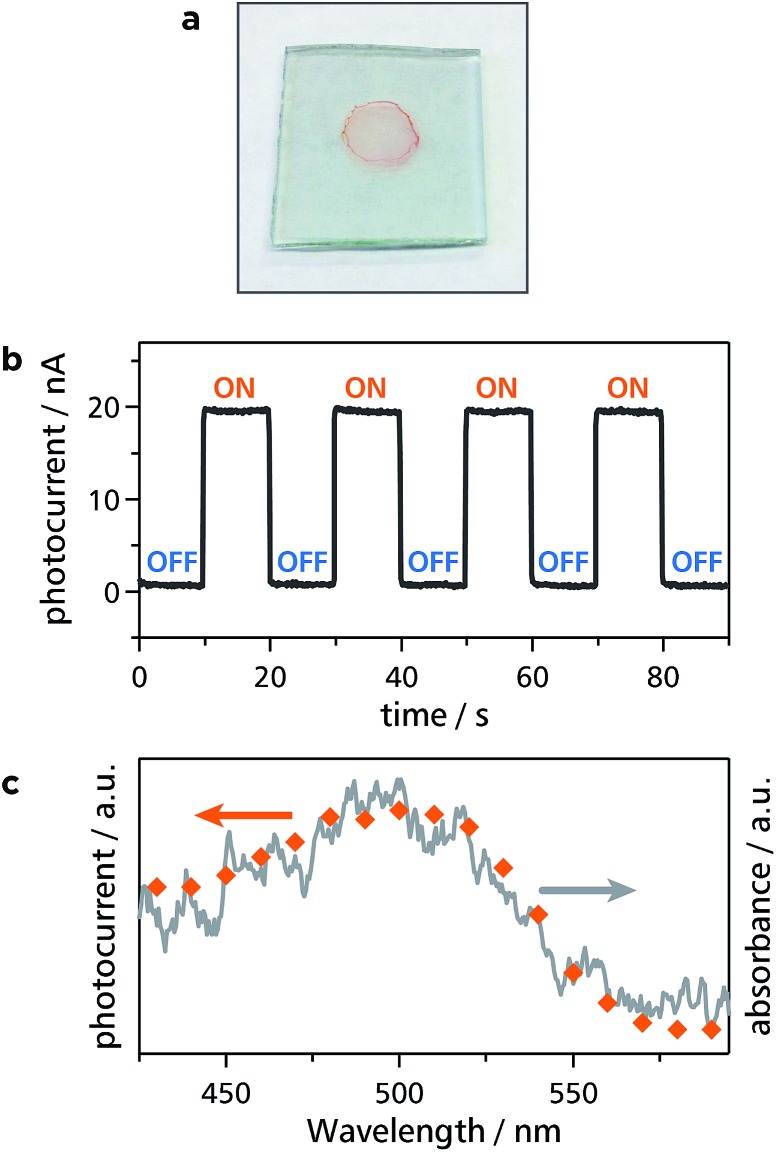
Photoelectric conversion employing **Zn1** as an active material. (a) Photograph of a thin film of **Zn1** on a SnO_2_ electrode. (b) Typical anodic photocurrent response upon irradiation of a working electrode (SnO_2_ substrate modified with **Zn1** as shown in (a)) with intermittent 500 nm light. (c) Action spectrum for the photocurrent generation (orange dots) and absorption spectrum of **Zn1** on a SnO_2_ substrate (gray solid line).

Thanks to intense light absorption disclosed in Fig. 2d and 5b, **Zn1** and **Zn2** are expected to show photofunctionality. As one of such demonstrations, **Zn1** and **Zn2** were built into a photoelectric conversion system. Dispersions of **Zn1** and **Zn2** were deposited onto transparent SnO_2_ electrodes, such that thin films of **Zn1** and **Zn2** were formed (Fig. 7a and S6a†): This also ensures the good processability of **Zn1** and **Zn2**. A three-electrode electrochemical cell was then set up (Fig. S7†), where the modified SnO_2_ electrode was employed as a photoanode. An anodic photocurrent was observed only when the **Zn1**-modified SnO_2_ electrode was illuminated with 500 nm light (Fig. 7b). In addition, the action spectrum for the photocurrent coincided with the absorption spectrum of **Zn1** on a SnO_2_ electrode (Fig. 7c). This series of facts indicates that **Zn1** functions as an active layer for the photoelectric conversion system. The authors then investigated the photoelectric conversion efficiency (Fig. S8 and S9†). As shown in Fig. S9a,† the photocurrent reaches the maximal value with the optical density of the film at 500 nm of ∼0.005. On the other hand, the quantum yield for the photoelectric conversion decreased as the growth of the film, showing the maximal value of 1.0% in an acetonitrile medium (Fig. S9b†). To demonstrate the predominance of **Zn1**, the authors also prepared mononuclear bis(dipyrrinato)zinc(ii) complex **Mono3** ^32^ with carboxy groups, which forms a self-assembled monolayer (SAM) on a SnO_2_ electrode upon chemisorption (Fig. S10a†). The SAM of **Mono3** performed a conversion efficiency of 0.069% in an acetonitrile medium (Fig. S10b and c†), which is much lower than that of **Zn1** with the same optical density. The **Zn2**-modified SnO_2_ electrode also served as a photoanode in the same manner as **Zn1**, except that the photoresponse is red-shifted, which is esteemed in photoelectric conversion applications (Fig. S6b and c†). The red-shift is induced by the π-extension of dipyrrin ligand **L2**, thereby demonstrating the designability and tunability of the present 1D-CPs. The highest conversion efficiency of **Zn2** (0.027% in an aqueous medium, Fig. S6d and S11†) is far greater than that of **Mono3** in the same medium (∼0%, Fig. S10d and e†). The series of comparative experiments displays that the polymer structure of **Zn1** and **Zn2** is advantageous for photoelectric conversion applications.

## Conclusions

The authors determined the structural and functional features of 1D-CPs comprising the bis(dipyrrinato)metal(ii) complex motif. Layering bridging dipyrrin ligand **L1** in dichloromethane and metal(ii) ions in water produced single crystals of 1D-CP **M1** through a spontaneous coordination reaction at the liquid/liquid interface. X-ray diffraction analysis showed that the fibers of **M1** were propagated along the [1 0 –1] crystallographic axis. Isolated fibers of **Zn1** could be exfoliated from the single crystal upon ultrasonication in dichloromethane, and AFM confirmed that the fibers were more than several μm long. **Zn1** did not show detectable fluorescence, whereas 1D-CPs **Zn2** comprising π-extended bridging dipyrrin ligand **L2** and zinc(ii) ions featured orange fluorescence with the maximum wavelength of 597 nm in a toluene dispersion: this demonstrated the designability and tunability of the present 1D-CP. The dispersibility of **Zn1** and **Zn2** was sufficient enough to afford a significant advantage in processing them for applications. **Zn1** formed a conjugate with SWCNTs, where the fibers of **Zn1** wrapped around SWCNTs uniformly, and which featured thermoelectric conversion. In addition, **Zn1** and **Zn2** could be deposited onto transparent SnO_2_ electrodes as thin films, which served as photoanodes for a photoelectric conversion system. The photoelectric conversion response could be extended to longer wavelengths by a dipyrrin ligand modification. Our present results herein highlight the utility of 1D-CPs in materials science.
