# Supplementary material for: Bis(dipyrrinato)metal(ii) coordination polymers: crystallization, exfoliation into single wires, and electric conversion ability
Source: Chem Sci. 2015 Feb 26;6(5):2853–8. doi: 10.1039/c5sc00273g (PMC5489023; doi:10.1039/c5sc00273g)
Supplement: Supplementary file 1 [file SC-006-C5SC00273G-s001.pdf]

Electronic Supplementary Material (ESI) for Chemical Science.
This journal is © The Royal Society of Chemistry 2015

Electronic Supplementary Information

Bis(dipyrrinato)metal(II) coordination polymers: crystallization, exfoliation into single wires, and electric conversion ability

Ryota Matsuoka,[a] Ryojun Toyoda,[a] Ryota Sakamoto,*[a] Mizuho Tsuchiya,[a] Ken Hoshiko,[a] Tatsuhiro Nagayama,[a] Yoshiyuki Nonoguchi,[b] Kunihisa Sugimoto,[c] Eiji Nishibori,[d] Tsuyoshi Kawai[b] and Hiroshi Nishihara*[a]

[a]Department of Chemistry, Graduate School of Science, The University of Tokyo, 7-3-1, Hongo, Bunkyo-ku, Tokyo 113-0033, Japan.
[b]Graduate School of Materials Science, Nara Institute of Science and Technology (NAIST), 8916-5 Takayama, Ikoma, Nara 630-0192, Japan.
[c]Japan Synchrotron Radiation Research Institute (JASRI), 1-1-1, Kouto, Sayo-cho, Sayo-gun, Hyogo 679-5198, Japan.
[d]Division of Physics, Faculty of Pure and Applied Sciences, Tsukuba Research Center for Interdisciplinary Materials Science (TIMS), and Center for Integrated Research in Fundamental Science and Engineering (CiRfSE), University of Tsukuba, 1-1-1 Tennodai, Tsukuba, Ibaraki 305-8571, Japan.

*e-mail: sakamoto@chem.s.u-tokyo.ac.jp, nisihara@chem.s.u-tokyo.ac.jp

S1 Experimental methods.
S2 XPS of **Zn1**, **Mono1**, **Ni1** and **Cu1**, and **Zn2**.
S3 Single crystals of **Ni1** and **Cu1**.
S4 ORTEP drawings of **Zn1**, **Ni1**, and **Cu1**.
S5 Crystallographic data of **Zn1**, **Ni1**, and **Cu1**.
S6 Free-standing film of **Zn1-SWCNT**.
S7 Thermoelectric conversion ability of **Zn1-SWCNT**.
S8 Photoelectric conversion ability of **Zn2** deposited on a transparent SnO_2 electrode.
S9 Photoelectric conversion setup.
S10 Determination of the quantum yield for the photoelectric conversion of **Zn1**.
S11 Relationship between the photoelectric conversion ability and the optical density of the film of **Zn1**.
S12 Determination of the quantum yield for the photoelectric conversion of **Mono3**.
S13 Determination of the quantum yield for the photoelectric conversion of **Zn2**.

S1 Experimental methods.

General experimental procedures. All chemicals were purchased from Tokyo Chemical Industry Co., Ltd., Kanto Chemical Co., or Wako Pure Chemical Industries, Ltd., unless otherwise stated. They were used without further purification, except for $Zn(OAc)_2{\cdot}2H_2O$, which was recrystallized from water. Water was purified using a Milli-Q purification system (Merck KGaA). HOPG was purchased from Alliance Biosystems, Inc. (Grade SPI-1, 10 × 10 × 2 mm) and was cleaved with adhesive tape just before use. Transparent SnO_2 electrode (on ITO-coated glass, 5 Ω sq^{-1}) was purchased from Geomatec co., ltd. It was sonicated in acetone (10 min), and nonionic detergent in water (30 min × 2). Then the substrate was washed with water till the bubble of the detergent disappeared, and sonicated in water (10 min). The cleaned substrate was stored in water, and dried by nitrogen blow just prior to use. **Mono2** and **Mono3** were synthesized according to the methods described previously.[S1] ^1H and ^{13}C NMR data were collected in $CDCl_3$, and were recorded on a Bruker DRX 500, a JEOL ECX-500, and a Bruker US500 spectrometer. Tetramethylsilane (δ_H = 0.00) was used as an internal standard for the ^1H NMR spectra, and $CDCl_3$ (δ_C = 77.00) was used as an internal standard for the ^{13}C NMR spectra. High-resolution fast-atom bombardment mass spectroscopy (HR-FAB-MS) was performed on a JEOL JMS-700 MStation mass spectrometer. XPS data were acquired using an ULVAC-PHI PHI 5000 VersaProbe spectrometer. Al Kα (15 kV, 25 W) was used as the X-ray source, and the beam was focused on a 100 μm^2 area. The spectra were analyzed using MultiPak Software and standardized using the C 1s peak at 284.6 eV. AFM measurements were carried out using an Agilent Technologies 5500 scanning probe microscope under ambient conditions in high-amplitude mode (tapping mode) with a silicon cantilever Nano World PPP-NCL probe. UV/vis spectra were recorded on a JASCO V-570 spectrometer. Luminescence spectra were collected with a HITACHI F-4500 spectrometer. Absolute photoluminescent quantum yields were measured by a Hamamatsu Photonics C9920-02G. TEM was conducted using a JEOL JEM-3100FEF. Raman spectra were recorded on a JASCO NRS-5100. SWCNTs (HP-grade, >80% carbon purity, diameter 1.0–1.4 nm, length 5–50 μm, semiconductor content >80%) were purchased from KH Chemicals Co., Ltd. All experiments were conducted under an ambient condition unless otherwise stated.

Synthesis of mononuclear bis(dipyrrinato)zinc(II) complex Mono1. Zinc(II) acetate (36.7 mg, 0.20 mol) and triethylamine (0.11 mL, 0.79 mmol) were added to a dichloromethane solution (20 mL) of 2-((3,5-dimethyl-2*H*-pyrrol-2-ylidene)(2,6-dimethylphenyl)methyl)-3,5-dimethyl-1*H*-pyrrole[S2] (122 mg, 0.40 mmol), and the reaction mixture was stirred overnight at room temperature. Methanol (20 mL) was added to recrystallize the product as an orange solid (60.5 mg, 45.0%). ^1H NMR (500 MHz, $CDCl_3$): δ = 7.21 (t, J = 7.6 Hz, 2H), 7.11 (d, J = 7.6 Hz, 4H), 5.91 (s, 4H), 2.16 (s, 12H), 2.04 (s, 12H), 1.28 (s, 12H); ^{13}C NMR (125 MHz, $CDCl_3$): δ = 156.07, 143.26, 143.06, 139.18, 135.95, 134.27, 127.98, 127.88, 119.74, 19.33, 16.13, 14.70; HR-FAB-MS: 670.3011 $[M]^+$, calcd. for: $C_{42}H_{46}N_4Zn^+$: 670.3014.

Synthesis of 5,5'-(2,3,5,6-tetramethyl-1,4-phenylene)bis((3,5-dimethyl-2*H*-pyrrol-2-ylidene)methylene))bis(2,4-dimethyl-1*H*-pyrrole), L1. Under a nitrogen atmosphere, 2,3,5,6-Tetramethylterephthalaldehyde (457 mg, 2.4 mmol), 2,4-dimethylpyrrole (1.0 mL, 9.7 mmol), and trifluoroacetic acid (10 μL) were added to dry dichloromethane (100 mL), and the resultant solution was stirred overnight in the dark at room temperature. Then, *p*-chloranil (1.18 g, 4.8 mmol) was added, and the reaction mixture was stirred for 2 h. After removal of the solvent under reduced pressure, the crude product was purified by alumina column chromatography (eluent: a mixture of hexane and dichloromethane (1 : 1 v/v), then dichloromethane). The yellow-orange band was collected, and evaporated under reduced pressure to give a brown powder, which was recrystallized further from dichloromethane and hexane to give **L1** as a brown-orange powder (642 mg, 50%). ^{1}H NMR (400 MHz, $CDCl_3$): δ = 13.89 (br, 2 H), 5.85 (s, 4 H), 2.36 (s, 12 H), 2.15 (s, 12 H), 1.56 (s, 12 H); ^{13}C NMR (100 MHz, $CDCl_3$): δ =151.1, 139.5, 139.0, 137.2, 136.0, 133.1, 118.7, 17.1, 16.1, 15.6; HR-FAB-MS: 531.3484 $[M+H]^+$, calcd. for: $C_{36}H_{43}N_4^+$: 531.3482.

Synthesis of one-dimensional bis(dipyrrinato)zinc(II) complex polymer Zn1 by means of a single-phase reaction. An ethanol solution of zinc(II) acetate (2.6 mg, 12 μmol) was added dropwise to a dichloromethane solution (25 mL) of **L1** (5.6 mg, 11 μmol) over a period of 30 min. After stirring for 1 d at room temperature, the resulting orange precipitate was filtered, washed with dichloromethane and ethanol, and dried under reduced pressure to give **Zn1** as a dark-orange powdery solid (5.0 mg, 80%). The formation of **Ni1** was confirmed by XPS, disclosing a nitrogen-to-zinc abundance ratio of 79.8 : 20.2 (calcd. 4 : 1, Fig. S1a).

Synthesis of one-dimensional bis(dipyrrinato)nickel(II) complex polymer Ni1 by means of a single-phase reaction. The same procedure as **Zn1** was employed, except for nickel(II) acetate as the metal source, to give a dark-red solid (3.0 mg, 51%). The formation of **Ni1** was confirmed by XPS, disclosing a nitrogen-to-nickel abundance ratio of 80.2 : 19.8 (calcd. 4 : 1, Fig. S1c).

Synthesis of one-dimensional bis(dipyrrinato)copper(II) complex polymer Cu1 by a single-phase reaction. The same procedure as **Zn1** was employed, except for copper(II) acetate as the metal source, to give a green solid (4.8 mg, 81%). The formation of **Cu1** was confirmed by XPS, disclosing a nitrogen-to-copper abundance ratio of 80.3 : 19.7 (calcd. 4 : 1, Fig. S1d).

Synthesis of one-dimensional bis(dipyrrinato)zinc(II) complex polymer Zn1 by means of a liquid/liquid interfacial reaction. **L1** (1.5 mg) was added to dichloromethane (10 mL) in a glass cylinder with a diameter of 40 mm, to obtain a solution with a concentration of 0.28 mM. The dichloromethane solution was then covered with pure water (10 mL), such that a two-phase system was formed. An aqueous solution (10 mL) of zinc(II) acetate (50 mM) was added gently to the water phase. The reaction system was

kept undisturbed for 30 d in the dark, and **Zn1** was observed as orange crystals floating on the interface, or sinking at the bottom of the reaction container. The crystals were collected by filtration.

Synthesis of one-dimensional bis(dipyrrinato)nickel(II) complex polymer Ni1 by means of a liquid/liquid interfacial reaction. The same procedure as **Zn1** was employed, except for nickel(II) acetate as the metal source, and 90 d as the reaction time.

Synthesis of bis(dipyrrinato)copper(II) complex polymer Cu1 by a liquid/liquid interfacial reaction. The same procedure as **Zn1** was employed, except for copper(II) acetate as the metal source, and 20 d as the reaction time.

Synthesis of 5,5'-(2,3,5,6-tetramethyl-1,4-phenylene)bis((3,5-dimethyl-4-iodo-2*H*-pyrrol-2-ylidene)methylene))bis(2,4-dimethyl-3-iodo-1*H*-pyrrole). To a solution of iodine (3.05 g, 12 mmol) in methanol (20 mL) were added iodic acid (2.16 g, 12.0 mmol) and **L1** (1.06 g, 2.0 mmol) dissolved in methanol (5 mL), and the mixture was stirred at room temperature until a red-brown solid precipitated from the mixture. After 100 mL of chloroform was added, the mixture was washed with aqueous sodium sulfite (100 mL × 2) followed by water (100 mL), and the organic phase was separated and dried over $MgSO_4$. The solvent was evaporated after the removal of $MgSO_4$, and the residue was recrystallized from chloroform and methanol to give the titled compound as a red-brown powder (1.54 g, 76%). ^{1}H NMR (500 MHz, $CDCl_3$): δ = 2.42 (s, 12H), 2.11 (s, 12H), 1.45 (s, 12H); ^{13}C NMR (125 MHz, $CDCl_3$): δ = 152.04, 141.06, 138.56, 137.35, 135.95, 133.39, 83.02, 17.57, 17.26, 17.12; HR-FAB-MS: 1033.9303 [M]$^+$, calcd for $C_{36}H_{38}I_4N_4^+$: 1033.9275.

Synthesis of 5,5'-(2,3,5,6-tetramethyl-1,4-phenylene)bis((3,5-dimethyl-4-(p-tolylethynyl)-2*H*-pyrrol-2-ylidene)methylene))bis(2,4-dimethyl-3-(p-tolylethynyl)-1*H*-pyrrole), L2. Under a nitrogen atmosphere, a mixture of 5,5'-(2,3,5,6-tetramethyl-1,4-phenylene)bis((3,5-dimethyl-4-iodo-2*H*-pyrrol-2-ylidene)methylene))bis(2,4-dimethyl-3-iodo-1*H*-pyrrole) (1.54 g, 1.5 mmol), $Pd(PPh_3)_2Cl_2$ (56 mg, 0.075mol), CuI (15 mg, 0.075 mmol), and 1-ethynyl-4-methylbenzene (1.2 mL, 9.5 mmol) in THF (35 mL) and triethylamine (15 mL) was heated at 70°C for 3 h. After removing the solvent, the crude product was passed through a short pad of alumina (eluent: dichloromethane). After evaporation of the solvent, the residue was purified by alumina column chromatography (eluent: hexane to remove low-polarity impurities, then the ratio of dichloromethane was increased gradually). The collected red band was evaporated to give **L2** as a dark red solid (1.14 g, 77%). ^{1}H NMR (500 MHz, $CDCl_3$): δ = 7.38 (d, 8H, J = 8.2 Hz), 7.13 (d, 8H, J = 7.6 Hz), 2.51 (s, 12H), 2.35 (s, 12H), 2.15 (s, 12H), 1.61 (s, 12H); ^{13}C NMR (125 MHz, $CDCl_3$): δ = 153.57, 140.78, 139.65, 137.78, 137.05, 136.17, 133.33, 131.21, 129.06, 120.92, 113.86, 95.84, 82.81, 21.48, 17.21, 15.17, 14.50; HR-FAB-MS: 986.5274 [M]$^+$, calcd for $C_{72}H_{66}N_4$: 986.5288.

Synthesis of π-extended bis(dipyrrinato)zinc(II) complex polymer Zn2. An ethanol solution (10 mL) of zinc(II) acetate (0.70 mg, 3.8 μmol) was added to a dichloromethane solution (20 mL) of **L2** (2.35 mg, 2.4 μmol). After stirring for 10 d at room temperature, the reaction mixture was evaporated. Reprecipitation from dichloromethane and hexane gave **Zn2** as a red powder (0.50 mg, 19%). The formation of **Zn2** was confirmed by XPS, disclosing a nitrogen-to-zinc abundance ratio of 79.4 : 20.6 (calcd. 4 : 1, Fig. S1e).

Synthesis of a conjugate of Zn1 and SWCNTs (Zn1-SWCNT). Crystals of **Zn1** (0.10 mg) and SWCNTs (1.0 mg) were suspended in DMF (10 mL) by ultrasonication (38 kHz, 80 W) for 90 min. The orange suspension was then shaken using a laboratory shaker (300 oscillations/min) for 24 h, which resulted in the disappearance of the orange color derived from **Zn1**: This change indicates that **Zn1** was adsorbed onto SWCNTs. The residue was filtered using a polytetrafluoroethylene membrane filter (pore size: 450 nm), washed thoroughly with DMF and dichloromethane to remove small molecules, and dried under a reduced pressure at 80°C, to produce a round, thin film of **Zn1-SWCNT** with diameter and thickness of 1 cm and 64 μm, respectively. A film of pristine SWCNTs was fabricated using the same method, with DMSO as a solvent and without **Zn1**, with diameter and thickness of 1 cm and 86 μm, respectively.

X-ray crystallography. Single-crystal X-ray diffractions for **M1** were collected at 100 K. The diffractions were recorded on a large cylindrical imaging plate with synchrotron radiation of λ = 0.7000 Å at SPring-8 beam line BL02B1 (Hyogo, Japan).[S3] The structures were solved by direct methods using SIR-92,[S4] and were refined by the full-matrix least-squares technique against F^2 implementing SHELXL-2013.[S5] CCDC 1012353 (for **Zn1**), 1044669 (for **Cu1**), and 1044670 (for **Ni1**) contain the crystallographic data for this paper. These data may be obtained free of charge from The Cambridge Crystallographic Data Centre via http://www.ccdc.cam.ac.uk/data_request/cif.

DFT calculation. In order to estimate the heights of **Zn1** and **Zn2**, DFT calculations for **Mono1** and **Mono2** were carried out. The Gausian 09 program[S6] was used for the geometrical optimization. The structures were optimized without any symmetry constraint. The B3LYP hybrid exchange-correlation functional[S7] was employed. The LanL2DZ basis set[S8] was used for Zn, and the 6-31G(d) basis set[S9] for the other atoms. Visualization of the result was performed using GaussView 5.0.8 software.[S10]

Thermoelectric conversion property. We had previously reported the thermoelectric conversion property of SWCNTs modified with small molecules,[S11] and the same procedure was employed here. The DC electrical conductivity was measured using a Mitsubishi Chemical Loresta GP MCP-T610 with the four-point probe method. The thermoelectric voltage was recorded using a Seebeck coefficient measurement system K20SB100-3R (MMR technology) equipped with a Joule-Thomson effect temperature controller. The buckypaper film was transferred onto the sample stage of the measurement system, and silver paste was used to create the electrical connection. The series of measurements was conducted at 310 K, or employ

310 K as the standard temperature.

Photoelectric conversion. A dispersion of **Zn1** in acetonitrile or **Zn2** in dichloromethane was dropcast on a transparent SnO_2 electrode, such that the coordination polymer was deposited in the range of a 5mmϕ circle. The polymers deposited on the SnO_2 electrodes were always subjected to UV/vis spectroscopy prior to photoelectric conversion. The baseline of the UV/vis spectra was corrected to exclude the effect of scattering caused by the film of **Zn1** and **Zn2**. The modified SnO_2 electrode was used as a working electrode (photoanode). As for **Zn1**, a home-made Ag^+/Ag reference electrode (0.01 M $AgClO_4$ in 0.1 M Bu_4NClO_4/acetonitrile) and a Pt wire counter electrode were implemented in order to establish a three-electrode system. The three electrodes were built into a photoelectrochemical cell shown in Fig. S7. The cell was filled with an acetonitrile solution of tetrabutylammonium perchlorate (0.1 M, as a supporting electrolyte) containing triethanolamine (TEOA, 0.05 M, as a sacrificial donor reagent). As for **Zn2**, the same photoelectrochemical system as that for **Zn1** was constructed except that aqueous sodium sulfate (0.1 M) containing TEOA (0.03 M) as an electrolyte solution and an Ag/AgCl reference electrode were used because of good dispersibility of **Zn2** in acetonitrile. The photoelectrochemical cell was sealed, and was deoxygenized by Ar bubbling for 5 min prior to the measurement. Monochromatic light for the action spectra shown in Fig. 7c and S6c (430–600 nm in every 10 nm) was extracted from a Xe lamp (MAX-302, Asahi Spectra Co., Ltd.), the photon flux of which was monochromated by a monochrometer (CT-10, JASCO Corporation). For the other photocurrent responses (e.g. Fig. 7b and S6b), monochromic light was provided by the Xe lamp equipped with a band-pass filter (500nm for **Zn1** and 550 nm for **Zn2**). The active area of the electrode was 0.264 cm^2, which was determined by a fluorocarbon rubber o-ring. The electrode potential was controlled using an electrochemical analyzer (ALS 750A, BAS Inc.). The potential of the photoanode was fixed at near the open circuit potential (–0.35 V vs Ag^+/Ag for **Zn1** and 0.15 V vs Ag/AgCl for **Zn2**) such that a negligible dark current was observed. The photocurrent was also collected through the electrochemical analyzer. The quantum yield for the photocurrent generation was calculated following a previous literature.[S12] A photon counter (8230E and 82311B, ADC Corporation) was employed to quantify the photon flux of the incident light. Referential mononuclear complex **Mono3** was immobilized on a SnO_2 electrode according to the method described previously.[S1b] The **Mono3**-modified SnO_2 electrode was subjected to photoelectric conversion using the same procedure for **Zn1** and **Zn2**.

S2 XPS of **Zn1**, **Mono1**, **Ni1**, **Cu1**, and **Zn2**.

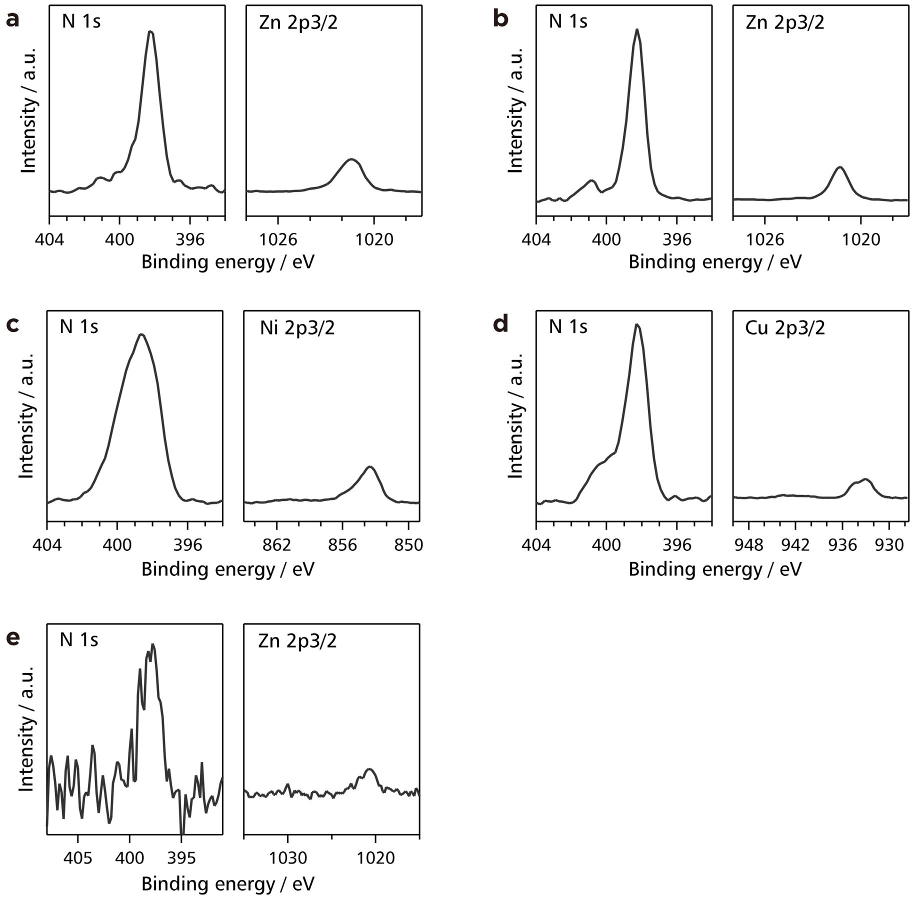

Fig. S1 XPS of (a) **Zn1** focusing on the N 1s and Zn 2p 3/2 regions, (b) **Mono1** focusing on the N 1s and Zn 2p 3/2 regions, (c) **Ni1** focusing on the N 1s and Ni 2p 3/2 regions, (d) **Cu1** focusing on the N 1s and Cu 2p 3/2 regions, and (e) **Zn2** focusing on the N 1s and Zn 2p 3/2 regions. The intensity is proportional to the element abundance: The original signal is standardized using the photoionization cross-section of each element.

S3 Single crystals of **Ni1** and **Cu1**.

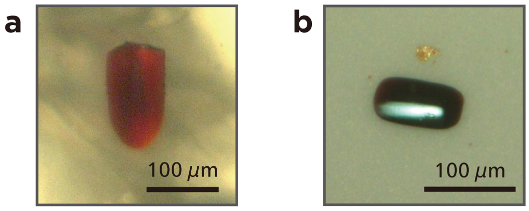

Fig. S2 Photographs of typical single crystals of (a) **Ni1** and (b) **Cu1**.

S4 ORTEP drawings of **Zn1**, **Ni1**, and **Cu1**.

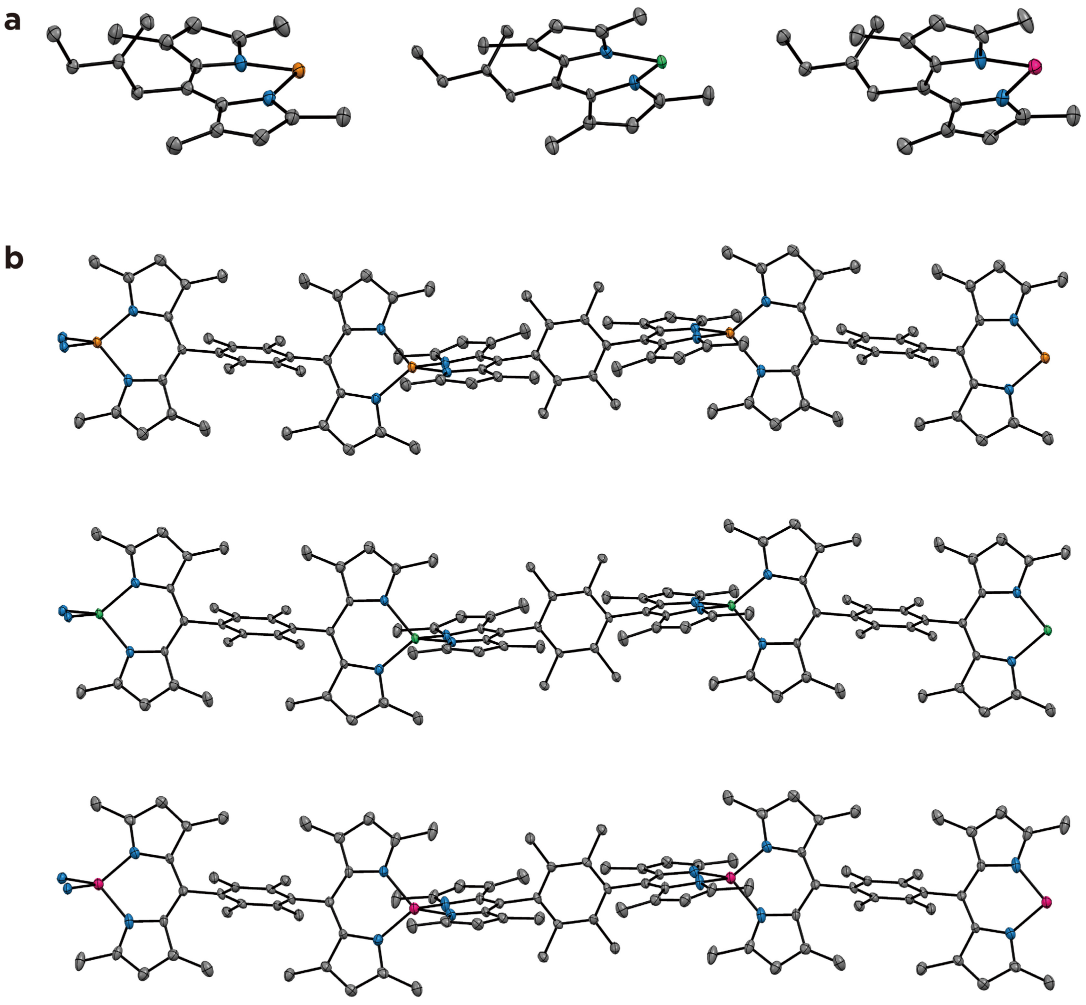

Fig. S3 (a) ORTEP drawings of **Zn1** (left), **Ni1** (center), and **Cu1** (right) showing the asymmetric unit. (C, gray; N, blue; Zn, orange; Ni, green; Cu, purple). (b) Perspective ORTEP drawings of **Zn1** (top), **Ni1** (middle), and **Cu1** (bottom) with a thermal ellipsoid was set at the 50% probability level. Hydrogen atoms are omitted for clarity.

S5 Crystallographic data of **Zn1**, **Ni1**, and **Cu1**.

Table S1 Crystallographic data of **Zn1**

Empirical formula	$C_{36}H_{40}N_4Zn_{0.5}$
Formula weight	561.40
Temperature	100(2) K
Wavelength	0.7000 Å
Crystal system	Monoclinic
Space group	C2/c (no. 15)
Unit cell dimensions	*a* = 18.8182(4) Å, *α* =90°
	b = 15.9892(3) Å, *β* = 114.760(8)°
	c = 10.4062(2) Å, *γ* =90°
Volume (*V*)	2843.26(19) $Å^3$
Z	4
Density (calculated)	1.311 g/cm^3
Absorption coefficient	0.430 mm^{-1}
F(000)	1196
Crystal size	$0.15 \times 0.07 \times 0.01$ mm^3
Theta range for data collection	2.317 to 35.683°
Index ranges	-37<=h<=30, -24<=k<=28, -18<=l<=20
Reflections collected	67,218
Independent reflections	6,635 (R_{int} = 0.0451)
Completeness to theta = 24.835°	100.0%
Refinement method	Full-matrix least-squares against F^2
Data / restraints / parameters	6635 / 0 / 190
Goodness-of-fit on F^2	0.995
Final *R* indices [$I > 2\sigma(I)$]	R_1 = 0.0529, wR_2 = 0.1394
R indices (all reflections)	R_1 = 0.0653, wR_2 = 0.1500
Largest diff. peak and hole	0.617 and -0.641 e$Å^{-3}$

Table S2 Crystallographic data of **Ni1**

Empirical formula	$C_{36}H_{40}N_4Ni_{0.5}$
Formula weight	558.07
Temperature	100(2) K
Wavelength	0.7000 Å
Crystal system	Monoclinic
Space group	C2/c (no. 15)
Unit cell dimensions	a = 18.8249(10) Å, α =90°
	b = 15.9650(10) Å, β = 114.696(8)°
	c = 10.4056(6) Å, γ =90°
Volume (V)	2841.3(3) $Å^3$
Z	4
Density (calculated)	1.305 g/cm^3
Absorption coefficient	0.343 mm^{-1}
F(000)	1192
Crystal size	0.07 × 0.06 × 0.01 mm^3
Theta range for data collection	1.72 to 27.5°
Index ranges	-34<=h<=26, -31<=k<=31, -15<=l<=20
Reflections collected	64,773
Independent reflections	3,297 (R_{int} = 0.0598)
Completeness to theta = 24.835°	98.4%
Refinement method	Full-matrix least-squares against F^2
Data / restraints / parameters	3297 / 0 / 189
Goodness-of-fit on F^2	1.129
Final R indices [I >2σ(I)]	R_1 = 0.0578, wR_2 = 0.1218
R indices (all reflections)	R_1 = 0.0629, wR_2 = 0.1291
Largest diff. peak and hole	0.535 and -0.589 $eÅ^{-3}$

Table S3 Crystallographic data of **Cu1**

Empirical formula	$C_{36}H_{40}N_4Cu_{0.5}$
Formula weight	560.49
Temperature	100(2) K
Wavelength	0.7000 Å
Crystal system	Monoclinic
Space group	C2/c (no. 15)
Unit cell dimensions	a = 18.8080(8) Å, α =90°
	b = 16.1023(7) Å, β = 115.201(8)°
	c = 10.3382(4) Å, γ =90°
Volume (V)	2832.9(3) $Å^3$
Z	4
Density (calculated)	1.314 g/cm^3
Absorption coefficient	0.386 mm^{-1}
F(000)	1194
Crystal size	0.09 × 0.07 × 0.01 mm^3
Theta range for data collection	1.715 to 27.496°
Index ranges	-31<=h<=31, -26<=k<=25, -17<=l<=17
Reflections collected	39,666
Independent reflections	3,319 (R_{int} = 0.0787)
Completeness to theta = 24.835°	98.9%
Refinement method	Full-matrix least-squares against F^2
Data / restraints / parameters	3319 / 0 / 189
Goodness-of-fit on F^2	0.891
Final R indices [$I > 2\sigma(I)$]	R_1 = 0.0570, wR_2 = 0.0975
R indices (all reflections)	R_1 = 0.0818, wR_2 = 0.1169
Largest diff. peak and hole	0.399 and -0.525 $eÅ^{-3}$

S6 Free-standing film of **Zn1-SWCNT**.

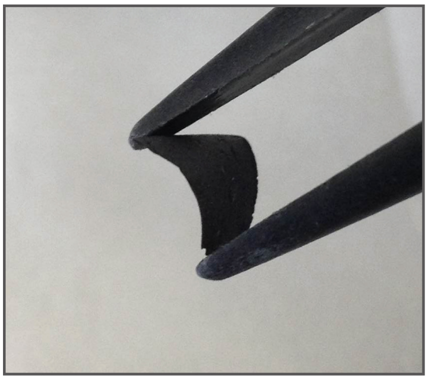

Fig. S4 Free-standing, flexible, round film of **Zn1-SWCNT** with a thickness of 64 µm.

S7 Thermoelectric conversion ability of **Zn1-SWCNT**.

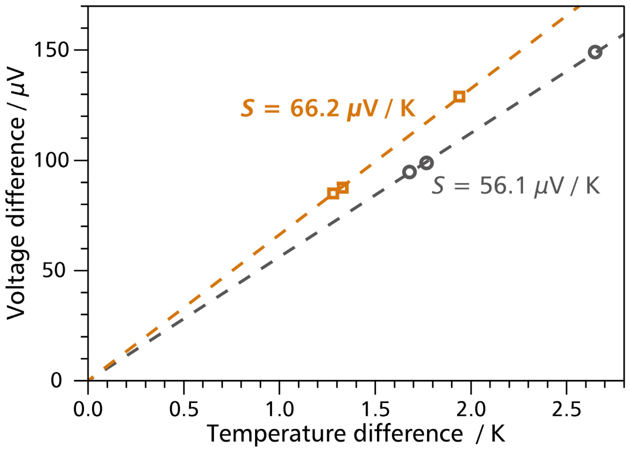

Fig. S5 Voltage-difference/temperature-difference plots for pristine SWCNTs (black) and **Zn1-SWCNT** (orange).

The conductivity of a film of pristine SWCNTs used herein possessed a positive Seebeck coefficient, *α*, of +56.1 μV K^{-1}. Thus, its electrical conductivity, *σ* (29.5 S cm^{-1}), is dominated by hole conduction (p-type semiconductor). On the other hand, a film of **Zn1-SWCNT** features greater *α* and *σ* values of +66.2 μV K^{-1} and 75.6 S cm^{-1}, respectively. As a result, **Zn1-SWCNT** has a power factor ($\alpha^2\sigma$, 33 μW m^{-1} K^{-2}) between three and four times greater than that of pristine SWCNTs (9.3 μW m^{-1} K^{-2}).

S8 Photoelectric conversion ability of **Zn2** deposited on a transparent SnO_2 electrode.

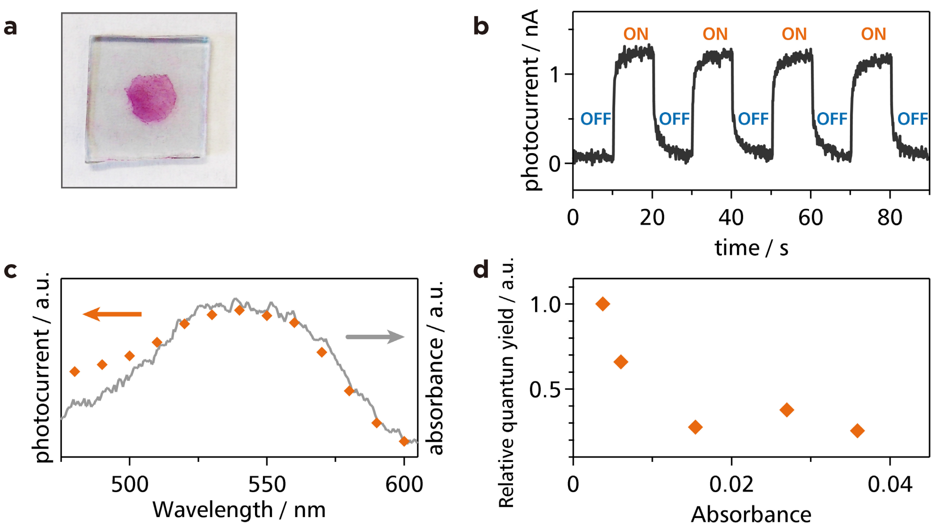

Fig. S6 Photoelectric conversion employing Zn2 as an active material. (a) Photograph of a thin film of **Zn2** on a SnO_2 electrode. (b) Typical anodic photocurrent response upon irradiation of a working electrode (SnO_2 substrate modified with **Zn2** as shown in (a)) with intermittent 550 nm light. (c) Action spectrum for the photocurrent generation (orange dots) and absorption spectrum of **Zn2** on a SnO_2 substrate (gray solid line). (d) Relationship between the relative quantum yield of the photoelectric conversion and absorbance of the film of **Zn2** upon irradiation with 550 nm light. The highest quantum yield (0.027%) is taken as the standard. Light intensity, 0.99 mW.

S9 Photoelectric conversion setup.

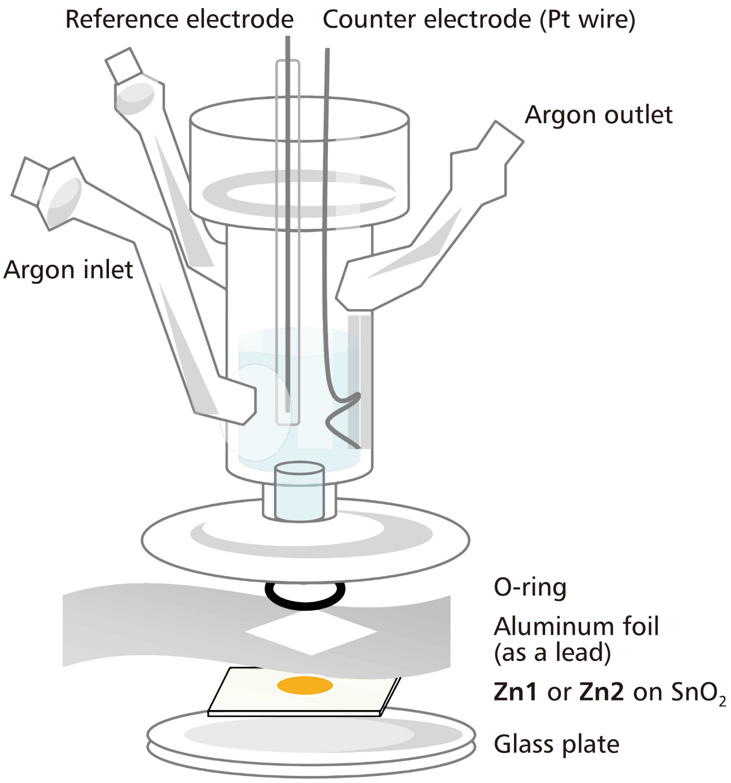

Fig. S7 Three-electrode electrochemical cell employed in the photocurrent generation. The components were clipped in order to ensure sealability. The incident light is illuminated in the direction vertical to the SnO_2 electrode (i.e. from the bottom of the cell).

S10 Determination of the quantum yield for the photoelectric conversion of **Zn1**.

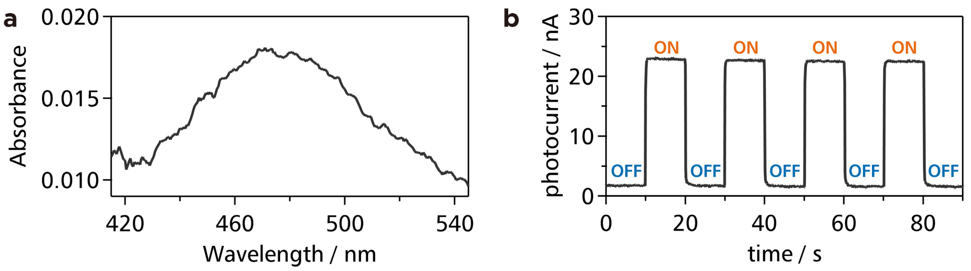

Fig. S8 (a) Typical absorption spectrum of **Zn1** deposited on a SnO_2 electrode. (b) Typical anodic photocurrent response upon irradiation of a working electrode (**Zn1**-modified SnO_2 electrode described in (a)) with intermittent 500 nm light in an acetonitrile medium. Light intensity, 0.90 mW. This measurement gave a quantum efficiency of 0.52%.

S11 Relationship between the photoelectric conversion ability and the optical density of the film of **Zn1**.

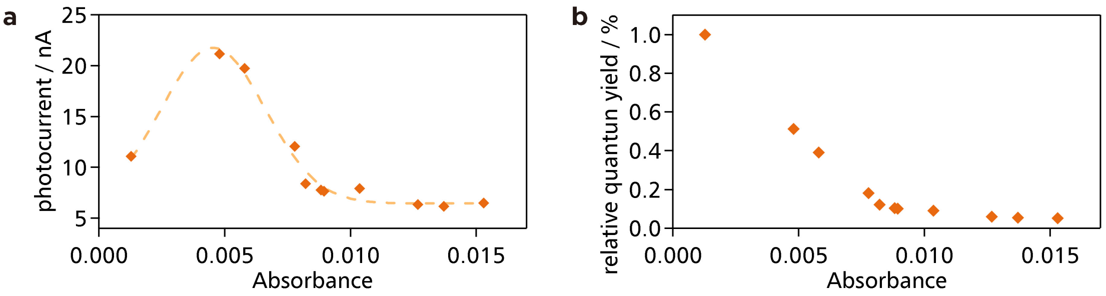

Fig. S9 Relationship between the absorbance of the film of **Zn1** and (a) photocurrent, (b) relative quantum yield of the photoelectric conversion upon irradiation with 500 nm light. The highest quantum yield (1.0%) is taken as the standard. Light intensity, 0.90 mW.

S12 Determination of the quantum yield for the photoelectric conversion of **Mono3**.

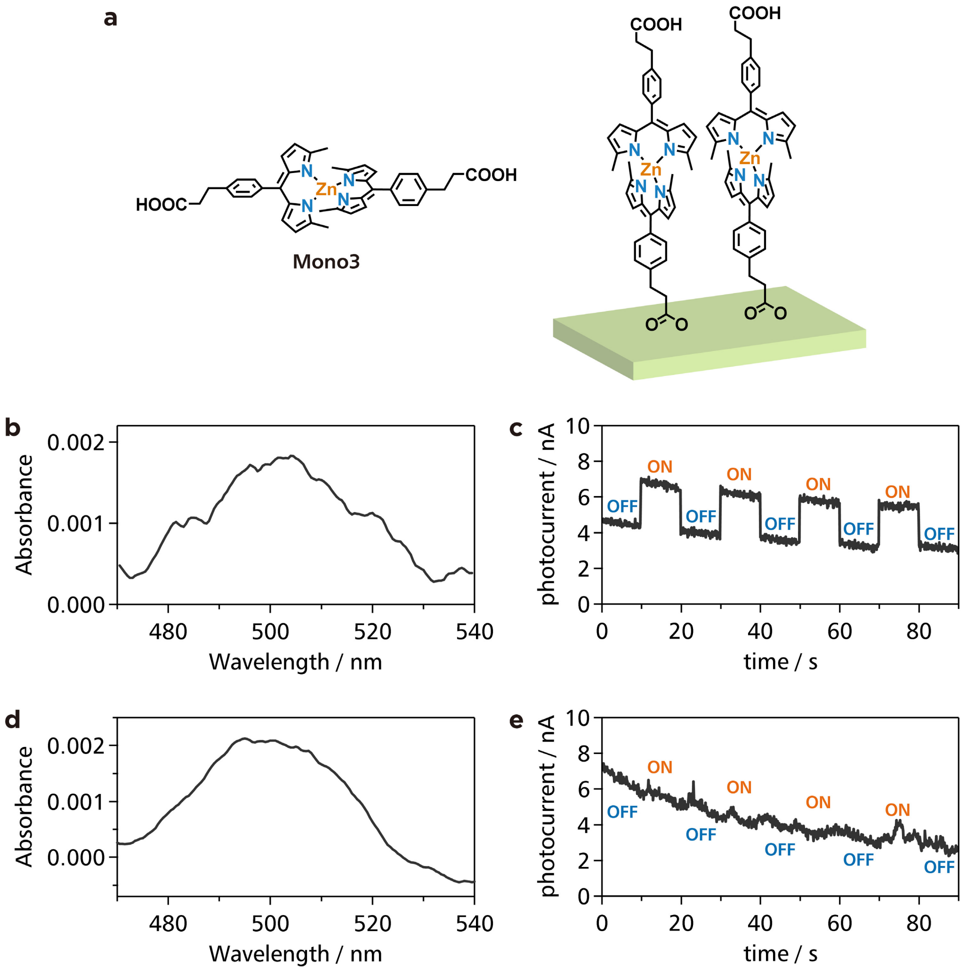

Fig. S10 (a) Chemical structure of referential mononuclear complex **Mono3**, and a schematic illustration on its self-assembled monolayer (SAM) on a SnO_2 electrode. (b) Absorption spectrum of **Mono3** chemisorbed as a SAM on a SnO_2 electrode. This electrode was used for the photocurrent measurement shown in (c). (c) Anodic photocurrent response upon irradiation of a working electrode (**Mono3**-modified SnO_2 electrode described in (b)) with intermittent 500 nm light in an acetonitrile medium. Light intensity, 1.8 mW. This measurement gave a quantum efficiency of 0.069%. (d) Absorption spectrum of **Mono3** chemisorbed as a SAM on a SnO_2 electrode. This electrode was used for the photocurrent measurement shown in (e). (e) Anodic photocurrent response upon irradiation of a working electrode (**Mono3**-modified SnO_2 electrode described in (d)) with intermittent 500 nm light in an aqueous medium. Light intensity, 1.8 mW. This photocurrent was so negligible that the quantum yield for the photoelectric conversion was regarded to be zero. (a)–(c) are adapted with permission from ref. S1b. Copyright 2015 Nature Publishing Group.

S13 Determination of the quantum yield for the photoelectric conversion of **Zn2**.

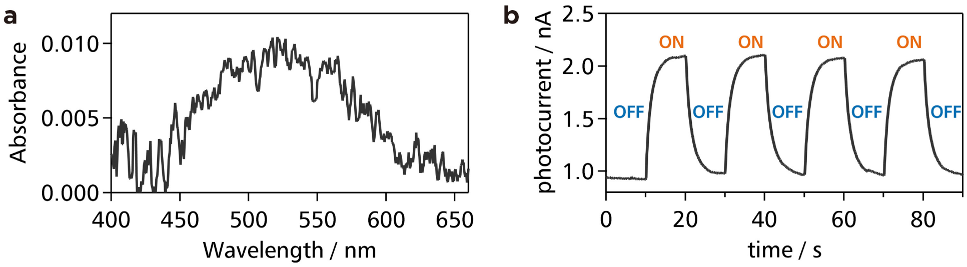

Fig. S11 (a) Typical absorption spectrum of **Zn2** deposited on a SnO_2 electrode. (b) Typical anodic photocurrent response upon irradiation of a working electrode (**Zn2**-modified SnO_2 electrode described in (a)) with intermittent 550 nm light in an aqueous medium. Light intensity, 0.99 mW. This measurement gave a quantum efficiency of 0.018%.

References for SI

S1. (a) S. Kusaka, R. Sakamoto, Y. Kitagawa, M. Okumura and H. Nishihara, *Chem. Asian J.*, 2012, **7**, 907. (b) R. Sakamoto, K. Hoshiko, Q. Liu, T. Yagi, T. Nagayama, S. Kusaka, M. Tsuchiya, Y. Kitagawa, W.-Y. Wong and H. Nishihara, *Nat. Commun.*, 2015, **6**, 6713.

S2. M. Tsuchiya, R. Sakamoto, S. Kusaka, Y. Kitagawa, M. Okumura and H. Nishihara, *Chem. Commun.* 2014, **50**, 5881.

S3. K. Sugimoto, H. Ohsumi, S. Aoyagi, E. Nishibori, C. Moriyoshi, Y. Kuroiwa, H. Sawa and M. Takata, *AIP Conf. Proc.,* 2010, **1234**, 887.

S4. A. Altomare, G. Cascarano, C. Giacovazzo, A. Guagliardi, M. C. Burla, G. Polidori and M. Camalli, *J. Appl. Crystallogr.*, 1994, **27**, 435.

S5. G. M. Sheldrick, *Acta Crystallogr. A.*, 2008, **64**, 112.

S6. M. J. Frisch, G. W. Trucks, H. B. Schlegel, G. E. Scuseria, M. A. Robb, J. R. Cheeseman, G. Scalmani, V. Barone, B. Mennucci, G. A. Petersson, H. Nakatsuji, M. Caricato, X. Li, H. P. Hratchian, A. F. Izmaylov, J. Bloino, G. Zheng, J. L. Sonnenberg, M. Hada, M. Ehara, K. Toyota, R. Fukuda, J. Hasegawa, M. Ishida, T. Nakajima, Y. Honda, O. Kitao, H. Nakai, T. Vreven, J. A. Montgomery, Jr., J. E. Peralta, F. Ogliaro, M. Bearpark, J. J. Heyd, E. Brothers, K. N. Kudin, V. N. Staroverov, R. Kobayashi, J. Normand, K. Raghavachari, A. Rendell, J. C. Burant, S. S. Iyengar, J. Tomasi, M. Cossi, N. Rega, J. M. Millam, M. Klene, J. E. Knox, J. B. Cross, V. Bakken, C. Adamo, J. Jaramillo, R. Gomperts, R. E. Stratmann, O. Yazyev, A. J. Austin, R. Cammi, C. Pomelli, J. W. Ochterski, R. L. Martin, K. Morokuma, V. G. Zakrzewski, G. A. Voth, P. Salvador, J. J. Dannenberg, S. Dapprich, A. D. Daniels, Ö. Farkas, J. B. Foresman, J. V. Ortiz, J. Cioslowski and D. J. Fox, Gaussian, Inc., Wallingford CT, **2009**.

S7. A. D. Becke, *J. Chem. Phys.*, 1993, **98**, 5648.

S8. P. J. Hay and W. R. Wadt, *J. Chem. Phys.*, 1985, **82**, 270.

S9. P. C. Hariharan and J. A. Pople, *Theor. Chim. Acta*, 1973, **28**, 213–222.

S10. Roy Dennington, Todd Keith and John Millam, *Semichem Inc.*, Shawnee Mission, KS, **2009**.

S11. Y. Nonoguchi, K. Ohashi, R. Kanazawa, K. Ashiba, K. Hata, T. Nakagawa, C. Adachi, T. Tanase and T. Kawai, *Sci. Rep.*, 2013, **3**, 3344.

S12. H. Yamada, H. Imahori, Y. Nishimura, I. Yamazaki and S. Fukuzumi, *Chem. Commun.*, 2000, 1921.
